# Molecular Recognition via Hydrogen Bonding in Supramolecular Complexes: A Fourier Transform Infrared Spectroscopy Study

**DOI:** 10.3390/molecules23092278

**Published:** 2018-09-06

**Authors:** Alfonso Martinez-Felipe, Fraser Brebner, Daniel Zaton, Alberto Concellon, Sara Ahmadi, Milagros Piñol, Luis Oriol

**Affiliations:** 1 Chemical and Materials Engineering Group, School of Engineering, University of Aberdeen, King’s College, Aberdeen AB24 3UE, UK; fraser.brebner.13@aberdeen.ac.uk (F.B.); r01dz17@abdn.ac.uk (D.Z.); 2 Department of Chemistry, School of Natural and Computing Sciences, University of Aberdeen, Meston Building, Old Aberdeen AB24 3UE, UK; 3 Departamento de Química Orgánica, Instituto de Ciencia de Materiales de Aragoón (ICMA)-Facultad de Ciencias, Universidad de Zaragoza-CSIC, 50009 Zaragoza, Spain; aconcellon@unizar.es (A.C.); mpinol@unizar.es (M.P.); loriol@unizar.es (L.O.); 4 Department of Chemistry, Firoozabad Branch, Islamic Azad University, 74715-117 Firoozabd, Iran; s.ahmadi@iauf.ac.ir

**Keywords:** supramolecular chemistry, hydrogen bonding, Fourier transform infrared spectroscopy, FT-IR, density functional theory, DFT

## Abstract

We assess the assembly of supramolecular complexes by hydrogen bonding between azocompounds and a diacylaminopyridine monomer by temperature-dependent Fourier transform infrared spectroscopy (FT-IR) and density functional theory (DFT) calculations. The electronic delocalisation in the supramolecular rings formed by multiple hydrogen bonds stabilises the complexes, which coexist with dimeric species in temperature-dependent equilibria. We show how the application of readily available molecular modelling and spectroscopic techniques can predict the stability of new supramolecular entities coexisting in equilibria, ultimately assessing the success of molecular recognition.

## 1. Introduction

Hydrogen bonding (HB) is at the core of the supramolecular chemistry involved in many natural processes [1]. It not only is the driving force to assemble the RNA and DNA helixes [2,3], but also plays a determining role in the transport of water and protonated species, such as crystallisation and dehydration of sugars [4,5] or the diffusion mechanisms of protons through fuel cell electrolytes [6,7,8,9]. HB is also a versatile tool to yield new synthetic complexes with tailor-made architectures [1,10,11,12,13,14,15,16,17]. The directionality and reversible character of the hydrogen bonds, for example, can assist the formation of supramolecular thermotropic liquid crystals by generating anisotropic structures tunable with temperature [18,19,20,21,22,23,24,25,26,27], including the formation of chiral ultrastructures from achiral molecules [28,29,30].

Hence, due to the ubiquitous nature of hydrogen bonds in condensed phases, effective molecular recognition between H-donors and H-acceptors represents a central challenge in the design of new supramolecular materials. Developing new methods and background to assess the formation of hydrogen bonds continues to have unmeasurable impact in different fundamental and applied fields.

Recently, we have prepared a series of supramolecular block copolymers with diacylaminopyridine units at the side chains, to which we attach molecules containing azobenzene groups by HB [31]. Our aim is to yield light-responsive materials for optical and light-controlled delivery applications with molecular design flexibility, and to avoid potential limitations derived from covalent-based post-functionalisation of the copolymers [32,33].

**Figure 1:**
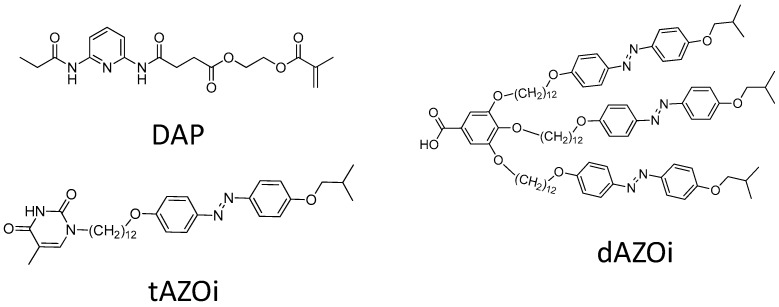
Molecular components in this study: dAZOi: Azo-dendron with benzoic acid termination; tAZOi: linear azo-compound with thymine head; DAP: the diacylaminopyridine monomer.

In this communication, we investigate the specific interactions between the molecular components similar to the repeating units of our derived macromolecules (Figure 1) by using Fourier transform infrared (FT-IR) spectroscopy [34], and Density Functional Theory (DFT) calculations [35]. Details on the materials preparation and the characterisation techniques can be found in Figure S2 in Supplementary Materials. We have labelled the dimers by the molecular component (DAP, dAZOi or tAZOi) in Figure 1, followed by the multiplicity of the hydrogen bonds formed (single, 1HB; double, 2HB; triple, 3HB) and the symmetry or asymmetry of the supramolecular structures (sym or as, respectively). Complexes are named by the molecular components involved separated by a dot, **●**, followed by the number of hydrogen bonds formed.

The FT-IR spectra of the three pristine compounds contain signs of HB, which are discussed in the next paragraphs for each compound (see Figure S2 in Supplementary Materials). More specifically, the appearance of several individual bands overlapped in the C=O (1750–1650 cm^−1^) and N-H (3600–3100 cm^−1^) IR stretching regions (*st*) reveals the coexistence of different supramolecular species in temperature-dependent equilibria [36]. The curves were fitted to Gaussian individual contributions, and the peak integrals were calculated and used to estimate the relative amounts of species present at different temperatures.

**Figure 2:**
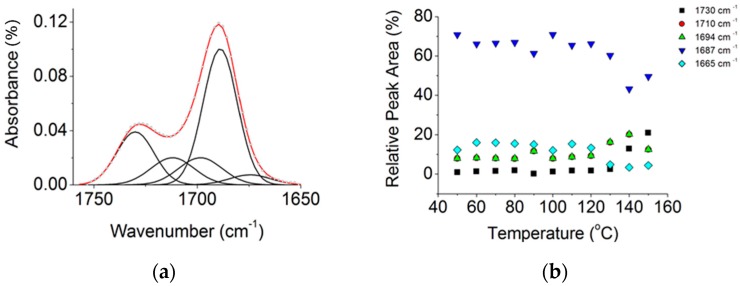
Temperature-dependent FT-IR spectra of dAZOi in the C=O *st.* region: (**a**) deconvolution of the region to different peaks at T=150 °C; (**b**) relative areas of the individual contributions as a function of temperature, on cooling.

**Figure 3:**
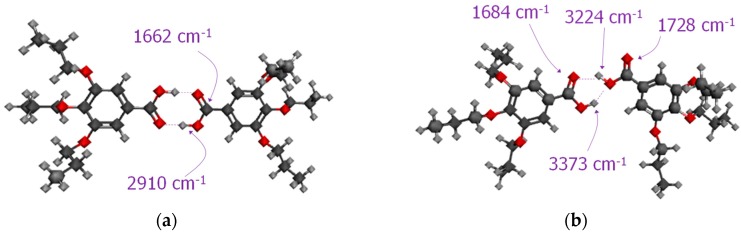
Models obtained by density functional theory (DFT) calculations corresponding to: (**a**) symmetric dAZOi dimers, dAZOi-2HB-sym, and (**b**) asymmetric dAZOi dimers, dAZOi-2HB-as, including the vibration frequencies calculated by DFT (see Table S1 in Supplementary Materials for associations with Figure 2). Dotted lines indicate hydrogen bonding.

Figure 2a shows the IR spectrum obtained at high temperatures for dAZOi in the C=O *st.* region, which was fitted to peaks associated to symmetric (1687 cm^−1^) and asymmetric dimers (1710 and 1694 cm^−1^) (see Figure 3), together with monomeric species (1730 cm^−1^) and catemeric aggregates (1665 cm^−1^) [37]. The formation of symmetric dimers formed by double hydrogen bonds are prevalent, dAZOi-2HB-sym, and their relative concentration increases on cooling (Figure 2b and Table S1 in Supplementary Materials). A strong band is predicted at values slightly higher than ~2900 cm^−1^ (Supplementary Materials, Figure S16), and can be detected by 2D-IR analysis (see Supplementary Materials, Figure S4), probably associated to the fundamental O-H stretching vibration [38].

**Figure 4:**
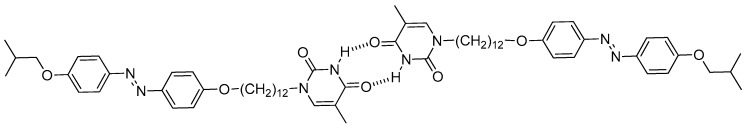
Proposed symmetric dimer formed between tAZOi units, tAZOi-2HB-sym. Dotted lines indicate hydrogen bonding.

The FT-IR spectra of tAZOi in the high frequency end is dominated by a main peak at 3156 cm^−1^, associated with N-H groups forming symmetric dimers via double hydrogen bonds with α,β-unsaturated C=O groups, tAZOi-2HB-sym (see Figure 4 and Figure S6 in Supplementary Materials) [39]. We cannot rule out, however, the formation of tAZOi open dimers assembled by single hydrogen bonds, acting as transitional states between different closed conformations (see Figure S8 in Supplementary Materials). One spectroscopic fingerprint of all these species is the presence of strong signals around ~1700 cm^−1^, arising from the simultaneous vibration of several groups of the thymine heads and the supramolecular ring. The potential stabilising role of the electronic delocalisation in these species will be discussed later.

**Figure 5:**
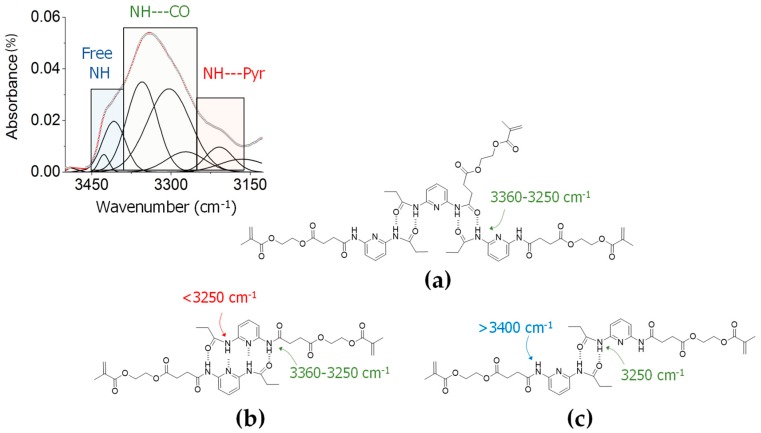
Schematic representations of different DAP supramolecular assemblies, including the N-H *st.* IR sub-regions for free N-H groups and those involved in hydrogen bonds with C=O groups and pyridine rings (Pyr): (**a**) DAP3-2HB-as, (**b**) DAP2-4HB-sym and (**c**) DAP2-2HB-sym.

Several DAP supramolecular assemblies are also predicted by DFT and are consistent with the broad IR N-H stretching region obtained for this sample (Figure S10 in Supplementary Materials). In addition to signals assigned to N-H free groups (>3400 cm^−1^), several bands appearing at higher frequencies (3360–3250 cm^−1^) can be associated with the formation of intermolecular hydrogen bonds between amide groups (N-H--C=O), whilst other signals at lower frequencies (<3250 cm^−1^) relate to N-H groups that are hydrogen bonded to the nitrogen atom in the DAP core (NH---N-Pyr). The lower-frequency bands increase on cooling, suggesting the preferential formation of DAP dimers assembled by a quadruple hydrogen bond, DAP2-4HB-sym, coexisting with other supramolecular species based on hydrogen bonds formed exclusively between amide groups, including a dimer, DAP2-2HB-sym, and a trimer, DAP3-2HB-as (see Figure 5).

**Figure 6:**
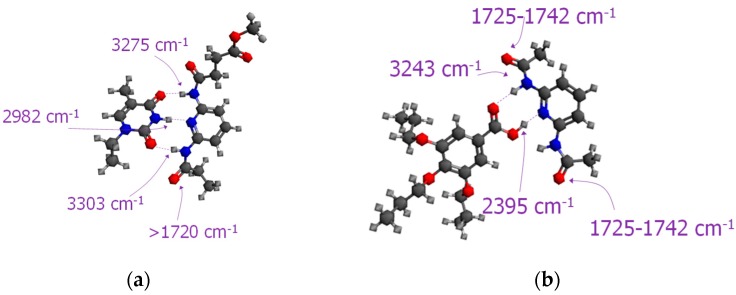
Formation of (**a**) DAP●tAZOi-3HB and (**b**) DAP●dAZOi-2HB hetero-complexes, via molecular recognition. Vibration frequencies calculated by DFT are also shown (see Table S4 in Supplementary Materials).

If we now turn our attention to the complexes, the experimental FT-IR spectra of the DAP+tAZOi mixture reveal the formation of hydrogen bonds by amide groups in DAP (~3277 and ~3214 cm^−1^), but the strong band peaked at 3156 cm^−1^ involved in the formation of tAZOi dimers is now considerably weaker (Figure S13 and Table S4 in Supplementary Materials). These observations, together with the appearance of new strong vibrational signals at lower frequencies, associated with new N-H---N-Pyr hydrogen bonds, suggest the formation of a hetero-complex assembled by a triple hydrogen bond, DAP●tAZOi-3HB, and these results are consistent with our DFT calculations (Figure 6a). The N-H stretching region of the DAP+dAZOi mixture, on the other hand, resembles that obtained for pristine DAP, showing three main individual peaks with maxima at 3352, 3310 and 3267 cm^−1^, respectively (see Figure S15 in Supplementary Materials). This result can be explained, at least in part, by the low complexation degree with dAZOi units in this mixture, which is kept at 30%, molar %, for the sake of consistency with previous works [31,32]. Several signals appear associated to the vibration of N-H groups in the DAP core that are hydrogen bonded to the carbonyl group of the benzoic acid, N-H---C=O (3264 cm^−1^), confirming the formation of the DAP●dAZOi-2HB complex illustrated in Figure 6b.

The stability of the different supramolecular dimers and complexes can be evaluated by comparing their theoretical dissociation energies, ∆E_dis_, obtained by sufficiently separating the individual molecules, and obtaining the difference in energy (Table 1) [37]. The formation of the DAP●tAZOi-3HB and DAP●dAZOi-2HB complexes is explained by their high dissociation energies, compared to most of the homomeric dimers, and their stabilities are also consistent with the weak temperature dependences of their IR signals (see Figure 2 and Figures S6, S10, S13 and S15 in Supplementary Materials). In the case of highly energetic dimers, nevertheless, the formation of the corresponding heterocomplexes via molecular recognition must be promoted by entropic contributions.

As expected, there is a positive correlation between the number of hydrogen bonds and their stability, but the ∆E_dis_ values do not follow a single additive rule in multiple hydrogen-bonded complexes. There is not much difference, for example, between the energies of DAP●tAZOi-3HB and DAP●dAZOi-2HB, despite the formation of one additional hydrogen bond in the former.

Indeed, the highest hydrogen-bonded unitary energy values correspond to the symmetric dimer dAZOi-2HB-sym, with around ∆E_dis_ ~ 59 kJ mol^−1^ per hydrogen bond. This result can be explained by the high strength of the hydrogen bonds formed between two benzoic acids, which are considered to have a symmetric charge distribution [40,41]. As the electronegativities of the H-acceptors and H-donor groups change, the symmetry of the charge distribution is reduced, with the strength of the hydrogen bond expected to decrease according to the sequence: O-H---O, N-H---O/O-H---N and N-H---N. This rule, however, must be taken carefully since may depend on the intermolecular environment of the compound, particularly in condensed phases, but is still consistent with the frequency displacements of the IR stretching signals obtained for the symmetric dimers of our pristine materials. For the O-H stretching vibration of **dAZOi-2HB-sym**, we calculated a frequency of ~2900 cm^−1^ (O-H---C=O), while the N-H stretching vibrations blueshift to ~3156 cm^−1^ for tAZOi-2HB-sym (N-H---O), and to ~3195 cm^−1^ for DAP2-4HB-sym (N-H---N-Pyr), respectively (see Tables S2 and S3 in Supplementary Materials).

Interestingly, the frequency value associated with the hydrogen bond between the nitrogen atom in the pyridine ring and the hydroxyl group in DAP●dAZOi-2HB is the lowest in this series, with 2395 cm^−1^ (O-H---N-Pyr) (Figure S26 in Supplementary Materials), compared with pristine benzoic acids and DAP dimers in the literature [42,43,44,45]. This result highlights the strong nucleophilic character of the nitrogen atom in the pyridine DAP core, and predicts the formation of stronger hydrogen bonds than those involving exclusively amide groups. The frequency value corresponding to the analogous N-H---N-Pyr vibration for DAP●tAZOi-3HB, on the other hand, is higher, due to the substitution of OH by NH, a weaker H-donor group.

**Figure 7:**
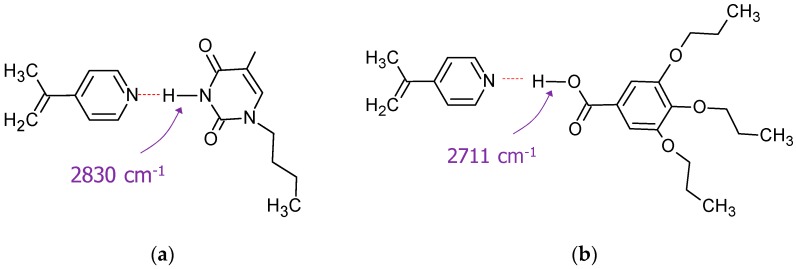
Complexes containing simple pyridine rings as H-acceptors: (**a**) Pyr●tAZOi-1HB and (**b**) Pyr●dAZOi-1HB. Vibration frequencies calculated by DFT are also shown. Dotted lines indicate hydrogen bonding.

These differences in individual HB strengths can explain why the stabilities of the two hetero-complexes seem to be very similar, despite having different number of hydrogen bonds. Indeed, pyridine and benzoic/carboxylic acids have been extensively used in the past to prepare a wide variety of supramolecular compounds [18,46,47,48,49]. For the sake of comparison, we have carried out similar DFT calculations on complexes containing dAZOi and tAZOi, but assembled via one single hydrogen bond with a pyridine monomeric ring (Figure 7). The dissociation energies are 62.83 kJ mol^−1^, for Pyr●tAZOi-1HB, and 74.82 kJ mol^−1^, for Pyr●dAZOi-1HB. As expected, these are lower ∆E_dis_ values than those obtained for our systems forming multiple hydrogen bonds, but not to a great extent. Indeed, these pyridine-based complexes exhibit the highest energies per hydrogen bond in the series. An interesting observation is that the corresponding O-H stretching vibration in Pyr●dAZOi-1HB appears at higher frequencies (2770 cm^−1^) than the value observed for DAP●dAZOi-2HB (~2400 cm^−1^). We believe that the use of the DAP core in our supramolecular systems, instead of a pyridine ring, may strengthen the HB formed with the O-H group by promoting resonating effects within the supramolecular ring. These are associated with strong IR signals around ~1700 cm^−1^ in the C=O stretching region of compounds forming multiple hydrogen bonds. The stabilising role of such delocalisation effects, and their IR assessment, is the focus of current investigation.

## 2. Conclusions

We have provided experimental and theoretical evidence on the formation of supramolecular complexes between diacylaminopyridine cores with thymine and benzoic acid groups, by arrays of multiple hydrogen bonds. The calculations predict that the complexes have lower dissociation energies than some of the dimers formed by the pristine compounds, coexisting in temperature-dependent equilibria of supramolecular species. The prominence of the hetero-complexes must be explained by entropic effects, and also by the formation of highly linear species, of which anisotropy may favour packing into crystalline phases [50].

Whilst the hydrogen bonds formed between O-H groups and the nitrogen atom in diacylaminopyridine and pyridine rings seem to be the strongest in this work, the presence of multiple hydrogen bonds may help stabilise the complexes by resonating effects occurring in the supramolecular rings. It is then arguable that similar complex stabilities could be achieved using a different substrate capable to form only two hydrogen bonds, for example, but provided with sufficient electrical delocalisation.

Even though the DFT calculations will never replicate the exact intermolecular environments of complexes formed within condensed phases, our results predict the stability of several new supramolecular entities assessed by FT-IR. Some of these predictions are indeed in excellent agreement with the frequency distribution of IR regions involving functional groups forming hydrogen bonds. More specifically, both DFT and FT-IR corroborate the formation of symmetric and asymmetric dAZOi dimers. To our knowledge, this is only the second report of this type of asymmetric benzoic acid dimer and provides further evidence of its formation [37].

We therefore believe that this methodology can contribute to create a library of systems that have the potential to form supramolecular complexes, by providing primary information on the conformations and stability of supramolecular species, and the success of molecular recognition. Some challenges in the field remain open for development, such as accounting for some of the resonance bands and other intermolecular forces in the condensed state, which may require the use of more powerful but also time-consuming molecular dynamic techniques.

## Figures and Tables

**Table 1:** Dissociation energies, ∆E_dis_/kJ mol^−1^, of the supramolecular dimers and trimers (modelling the pristine compounds) and complexes (modelling the mixtures) obtained by DFT.

**Dimers (and DAP Trimer)**			
dAZO_i_-2HB-sym		dAZO_i_-2HB-as	
116.91		60.23	
tAZO_i_-2HB-sym	tAZO_i_-2HB-sym(alt)	tAZO_i_-2HB-as	tAZO_i_-1HB-as
76.58	70.81	73.15	43.01
DAP2-4HB-sym	DAP3-2HB-as		DAP2-2HB-sym
118.21	129.45		83.35
**Complexes**			
DAP●tAZOi-3HB		DAP●dAZOi-2HB	
92.92		89.66	
Pyr●tAZOi-1HB		Pyr●dAZOi-1HB	
62.83		74.82	

## Supplementary Materials

The following are available online. Figure S1: materials preparation, Figures S2 to S15: FT-IR results and proposed models by DFT, Figures S16 to S28: theoretical IR spectra, Tables S1 to S4: experimental and predicted IR frequencies, Table S5: lengths of the hydrogen bonds estimated by DFT.
