# Supplementary material for: Molecular Recognition via Hydrogen Bonding in Supramolecular Complexes: A Fourier Transform Infrared Spectroscopy Study
Source: Molecules. 2018 Sep 6;23(9):2278. doi: 10.3390/molecules23092278 (PMC6225378; doi:10.3390/molecules23092278)
Supplement: Supplementary file 1 [file molecules-23-02278-s001.pdf]

Communication

Molecular recognition *via* hydrogen bonding in supramolecular complexes: a Fourier transform infrared spectroscopy study

Supplementary Information

Alfonso Martinez-Felipe[1,*], Fraser Brebner[1], Daniel Zatón[1,2], Alberto Concellon[3], Sara Ahmadi[4], Milagros Piñol[3] and Luis Oriol[3]

1 Chemical and Materials Engineering Group, School of Engineering, University of Aberdeen. King's College, Aberdeen AB24 3UE, UK; a.martinez-felipe@abdn.ac.uk, fraser.brebner.13@aberdeen.ac.uk., r01dz17@abdn.ac.uk.

2 Department of Chemistry, School of Natural and Computing Sciences, University of Aberdeen, Meston Building, Old Aberdeen AB24 3UE, UK;

3 Departamento de Química Orgánica, Instituto de Ciencia de Materiales de Aragón (ICMA)-Facultad de Ciencias, Universidad de Zaragoza-CSIC, 50009, Zaragoza, Spain; aconcellon@unizar.es, mpinol@unizar.es, loriol@unizar.es.

4 Department of Chemistry, Firoozabad Branch, Islamic Azad University, Firoozabd, Iran; s.ahmadi@iauf.ac.ir.

* Correspondence: a.martinez-felipe@abdn.ac.uk; Tel.: +44(0) 1224 273 074.

CONTENTS

1. Materials preparation, **Fig. S1**………………………………………………….. 2
2. Characterisation techniques…………………………………………………….. 4
3. Additional figures, **Figures S2** to **S15**..……………………………...………... 6
4. Additional tables, **Tables S1** to **S5**..…………………………….……….. 13
5. Theoretical IR spectra calculated by DFT, **Figures S16** to **S28**……………... 18
6. References………………………………………………………………………. 25

1. Materials preparation

The three components in our supramolecular systems are the azocompounds 3,4,5-tris[12-(4-(4′-isobutyloxyphenyldiazo)phenoxy)dodecyloxy)]benzoic acid, **dAZOi**, and N(1)-[12-(4-(4′-isobutyloxyphenyldiazo)phenoxy)dodecyloxy)]thymine, **tAZOi**, and the monomer 2-(propionylamino)-6-[4-(2-methacryloyloxy ethoxy)-4-oxobutanoylamino]pyridine, **DAP**, see Figure S1.

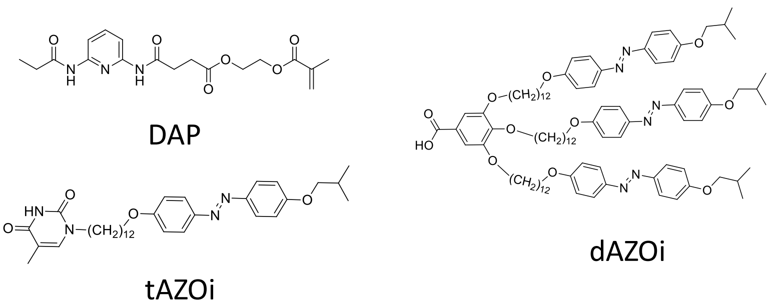

Figure S1. . Azo-dendron with benzoic acid termination, **dAZOi**, linear azo-compound with thymine head, **tAZOi**; the diaminopyridine monomer, **DAP**, used for polymerisation into **PDAP.**

DAP [1], **tAZOi** [2] and **dAZOi** [2] were previously synthesised, and their corresponding characterisation data are summarised below. 6-Diaminopyridine (Sigma-Aldrich, 98%) was recrystallized in ethanol before use. All other commercially available starting materials were purchased from Sigma-Aldrich and used as received without further purification. The mixtures, **dAZOi+DAP** and **tAZOi+DAP**, were prepared by mixing appropriate amounts of the individual compounds in dichloromethane, with stirring overnight at room temperature, and then allowing to evaporate during five days. The resulting powders were then further dried under vacuum for 24 hours. While the mixture **tAZOi+DAP** was aimed for a complete complexation (similar equivalents of **tAZOi** and **DAP** units were used), a 30% complexation of **DAP** units with **dAZOi** was targeted for **dAZOi+DAP**, in order to

achieve similar hydrophilic to hydrophobic ratios, and for the sake of consistency with previous works [2].

Room temperature Fourier transform infrared spectra, FT-IR, were obtained on a Bruker Vertex 70 FT-IR spectrophotometer using KBr pellets. Solution NMR experiments were carried out on Bruker Avance spectrometers operating at 400 MHz for 1H and 100 MHz for 13C, using standard pulse sequences. Chemical shifts are given in ppm relative to TMS and this was used as internal reference. Elemental analysis was performed using a Perkin-Elmer 2400 microanalyzer. MALDI-TOF MS was performed on an Autoflex mass spectrometer (Bruker Daltonics) using dithranol as matrix.

Characterisation data of dAZO$_i$

IR (KBr) ν (cm^{-1}): 1688, 1581, 1500, 1471, 1241, 1147, 842. ^{1}H-NMR (400 MHz, $CDCl_3$) δ (ppm): 7.94–7.78 (m, 12H), 7.31 (s, 2H), 7.05–6.88 (m, 12H), 4.05–3.91 (m, 12H), 3.77 (d, *J*=6.6 Hz, 6H), 2.17–2.05 (m, 1H), 1.86–1.67 (m, 12H), 1.51–1.17 (m, 48H), 1.02 (d, *J*=6.6 Hz, 18H). ^{13}C-NMR (100 MHz, $CDCl_3$) δ (ppm): 170.7, 161.4, 153.0, 147.1, 143.26, 124.4, 123.7, 114.8, 114.8, 108.7, 74.8, 73.7, 69.4, 68.5, 29.8, 29.6, 29.5, 29.4, 28.4, 26.2, 26.2, 19.4. MS (MALDI$^+$, dithranol, m/z): calcd. for $C_{91}H_{126}N_6O_{11}$, 1478.95; found, 1480.01 [M+1]$^+$, 1502.02 [M+Na]$^+$. Anal. calcd. for $C_{91}H_{126}N_6O_{11}$: C, 73.85%; H, 8.58%; N, 5.68%. Found: C, 73.83%; H, 9.00%; N, 5.53%. M.p.: 141ºC.

Characterisation data of **tAZO$_i$**

IR (KBr) ν (cm^{-1}): 3154, 3032, 1681, 1601, 1580, 1499, 1471, 1241, 1145, 838. ^{1}H-NMR (400 MHz, $CDCl_3$) δ (ppm): 8.59 (s, 1H), 7.92–7.80 (m, 4H), 7.03–6.91 (m, 4H), 3.97 (t, *J* = 6.6 Hz, 2H,), 3.74 (d, *J* = 6.7 Hz, 2H), 3.65–3.57 (m, 2H), 2.17–2.06 (m, 1H), 1.83 (d, *J* = 1.1 Hz, 3H), 1.77–1.66 (m, 2H), 1.42–1.16 (m, 16H), 0.99 (d, *J* = 6.6 Hz, 6H). ^{13}C-NMR (100 MHz, $CDCl_3$) δ (ppm): 164.2, 161.4, 161.3, 150.8, 147.1, 140.5, 124.4, 114.8, 110.6, 74.8, 68.4, 48.7, 29.6, 29.3, 26.1, 22.2, 19.4, 12.5. MS (MALDI$^+$, dithranol, m/z): calcd. for $C_{33}H_{46}N_4O_4$, 562.35; found, 563.37 [M+1]$^+$, 585.36 [M+Na]$^+$. Anal. calcd. for $C_{33}H_{46}N_4O_4$: C, 70.43%; H, 8.24%; N, 9.96%. Found: C, 70.04%; H, 8.29%; N, 9.81%. M.p.: 166ºC.

Characterisation data of **DAP**

IR (KBr) ν (cm^{-1}): 3336, 1724, 1690, 1639, 1586, 1512, 1453, 1316, 1152, 805. ^{1}H-NMR ($CDCl_3$, 400 MHz) δ (ppm): 8.10–7.60 (m, 5H), 6.14–6.09 (m, 1H), 5.60–5.53 (m, 1H), 4.44–4.30 (m, 4H), 2.85–2.74 (m, 2H), 2.72–2.63 (m, 2H), 2.43 (q, 2H, J= 7.5 Hz), 1.98–1.88 (m, 3H), 1.24 (t, 3H, J= 7.5 Hz). ^{13}C-NMR ($CDCl_3$, 100 MHz) δ (ppm): 172.6, 172.5, 167.4, 149.8, 149.3, 140.9, 136.0, 126.4, 109.6, 109.5, 62.7, 62.5, 32.3, 30.8, 29.4, 18.4, 9.5. MS (MALDI$^+$, dithranol, m/z): calcd. for $C_{18}H_{23}N_3O_6$, 377.16; found, 378.17 [M+1]$^+$, 400.15 [M+Na]$^+$. Anal. calcd. for $C_{18}H_{23}N_3O_6$: C, 57.29%; H, 6.14%; N, 11.13%. Found: C, 57.32%; H, 6.31%; N, 11.20%. M.p.: 123ºC.

2. Characterisation techniques

The temperature dependent FT-IR spectra were obtained using a Thermo Nicolet 470 FT-IR spectrometer over a frequency range 4000-400 cm^{-1}, with an accuracy of 4 cm^{-1}, as the average of 64 scans. The experiments were carried out in transmittance mode, on dispersions of the samples in potassium bromide, KBr, of 1% in weight. Homogeneous discs were obtained by grinding the corresponding amounts of sample and KBr into fine powder, and then pressing at around 7 atm for 5 min. A background measurement was taken at room temperature on a pristine KBr disc, and was subtracted to the sample spectra. Temperature was controlled by placing the discs into a Linkam TMS93 heating stage, with a temperature accuracy of ±0.1. Samples were heated to 150°C and then cooled down to room temperature. The IR spectra were then measured on isothermal steps every 5 or 10°C, allowing the sample to equilibrate at each temperature before measuring.

Additionally, we have also applied static 2D correlation spectroscopy to some of the experimental IR spectra [3-5] in order to detect cross correlation of spectral intensity variations with the temperature [6]. 2D correlation spectroscopy can discriminate potential overlapping peaks, and spreads these over the second dimension, in synchronous and asynchronous plots. The synchronous spectrum $\phi(\nu_1, \nu_2)$ signifies the simultaneous change in spectral intensities seen at the two different wavenumbers ν_1, and ν_2. If the spectral intensities at the two wavenumbers change in the same direction then the synchronous correlation sign will positive. If one is decreasing and the other increasing the sign will be negative. In case that $\phi(\nu_1, \nu_2) = 0$, then the order of intensity variations is impossible to determine. The asynchronous spectrum $\Psi(\nu_1, \nu_2)$, alternatively, represents the out of phase changes of spectral intensities. When $\Psi(\nu_1, \nu_2) = 0$ then the variations of spectral intensities at the two wavenumbers are fully synchronized. If $\phi(\nu_1, \nu_2)$and $\Psi(\nu_1, \nu_2)$ have the same sign then at wavenumber υ_1, spectral intensity variations occur before they do at wavenumber υ_2. If the signs are different, then the opposite occurs.

The theoretical conformations of the supramolecular systems were obtained by density functional theory, DFT, using simplified molecular models of our compounds. We have used the Gaussian 09 package [7], and more particularly the hybrid B3LYP functional. We have estimated the best minimum energy conformations by full geometrical optimisation, and obtained the theoretical infrared spectroscopy response of the conformers in the gas phase. The use of a diffusion function in a large basis set in this work, B3LYP/6-311++g** [8], allows the application of the original theoretical frequencies obtained by DFT, with only small deviations [9, 10]. Moreover, considering that the scaling factor for our method and basis set is 0.960 in the high frequency range and 0.988 below 1800 cm^{-1} [11], we believe that the corrections would fall within the error of not considering the intermolecular environment of the complexes, which is inherent of condensed phases. Therefore, we consider our

calculations to being valid to explain hydrogen bonding, but we note that the frequencies must be treated carefully, due to inherent deviations between gas and condensed phases.

In dimers and complexes, we have also calculated the dissociation energies, ΔE_{dis}, by sufficiently separating the individual molecules and obtaining the difference in energy. We justify the use of simplified DFT models of the compounds as a good approximation to study hydrogen bonding in the complexes, since the local environment of the groups involved are similar to those in the fully developed structures.

3. Additional figures

Figure S2 shows an overview of the FT-IR spectra of the three pristine materials [12].

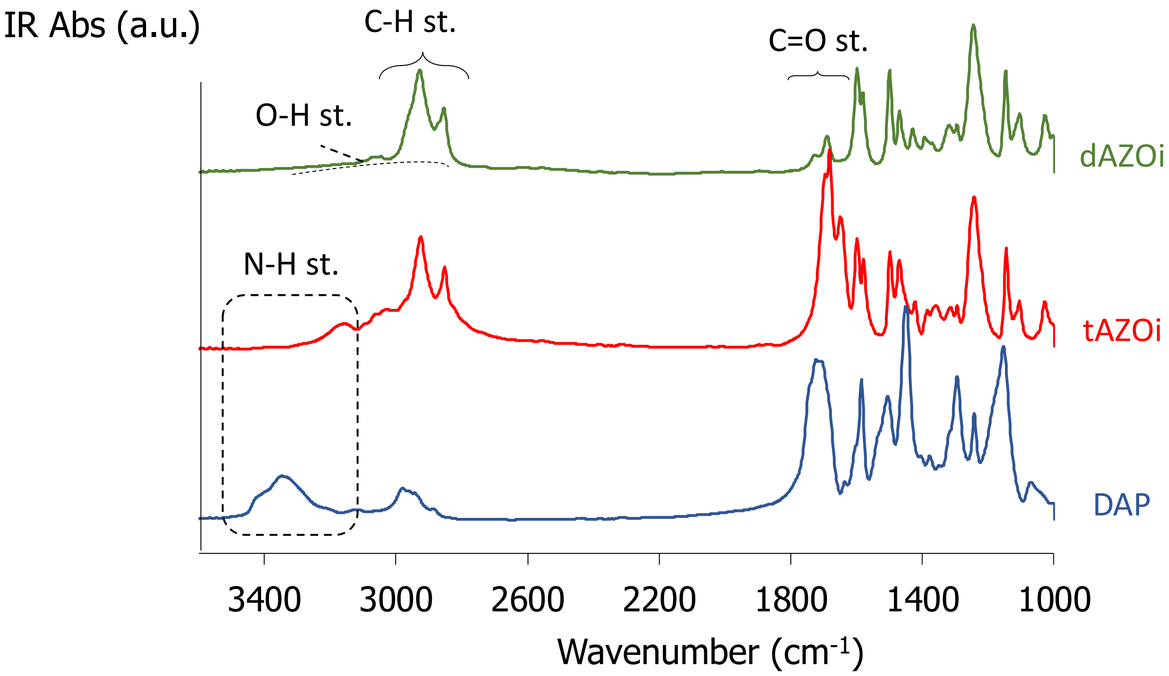

Figure S2. FT-IR spectra corresponding to the pristine compounds under study, obtained at $T = 150$°C. Curves have been shifted along the Y-axis arbitrarily.

Figures S3 to S11 show 3D plots corresponding to the temperature dependence of the FT-IR response of the pristine compounds, **dAZOi**, **tAZOi** and **DAP**, as well as examples of fittings at high temperature and the corresponding evolution of the individual peaks areas on cooling. Some additional DFT models of supramolecular species are also shown.

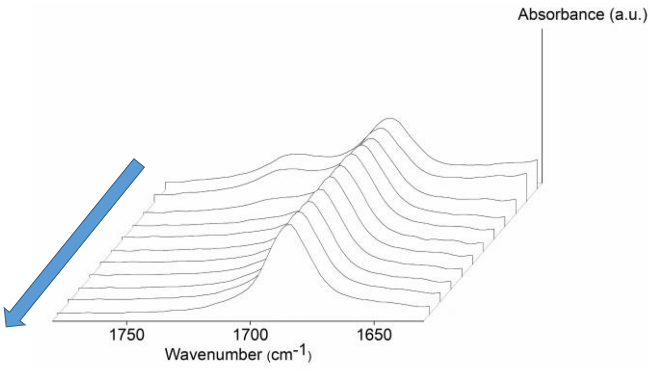

Figure S3. 3D-plot showing the temperature dependence of the FT-IR spectra of **dAZOi**, in the C=O *st.* region. Arrow indicates the direction on cooling.

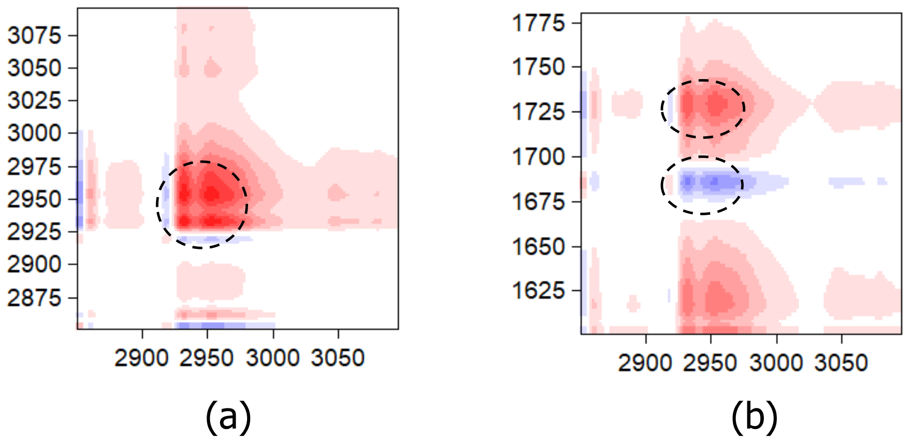

Figure S4. IR 2D synchronous plots obtained for **dAZOi**: **(a)** O-H stretching region and **(b)** cross-correlation between the C=O and O-H stretching regions. All axes display wavenumbers, in cm^{-1}. The strong correlations between ~2910 cm^{-1} and the C=O stretching region, confirm the participation of the hydroxyl groups to form the dAZOi dimers.

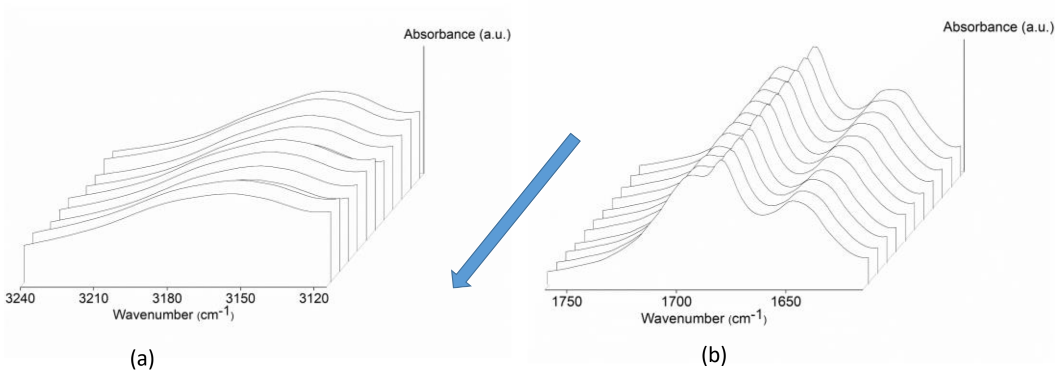

Figure S5. 3D-plots showing the temperature dependence of the FT-IR spectra of **tAZOi**, in the **(a)** N-H *st.* and **(b)** C=O *st.* regions. Arrow indicates the direction on cooling.

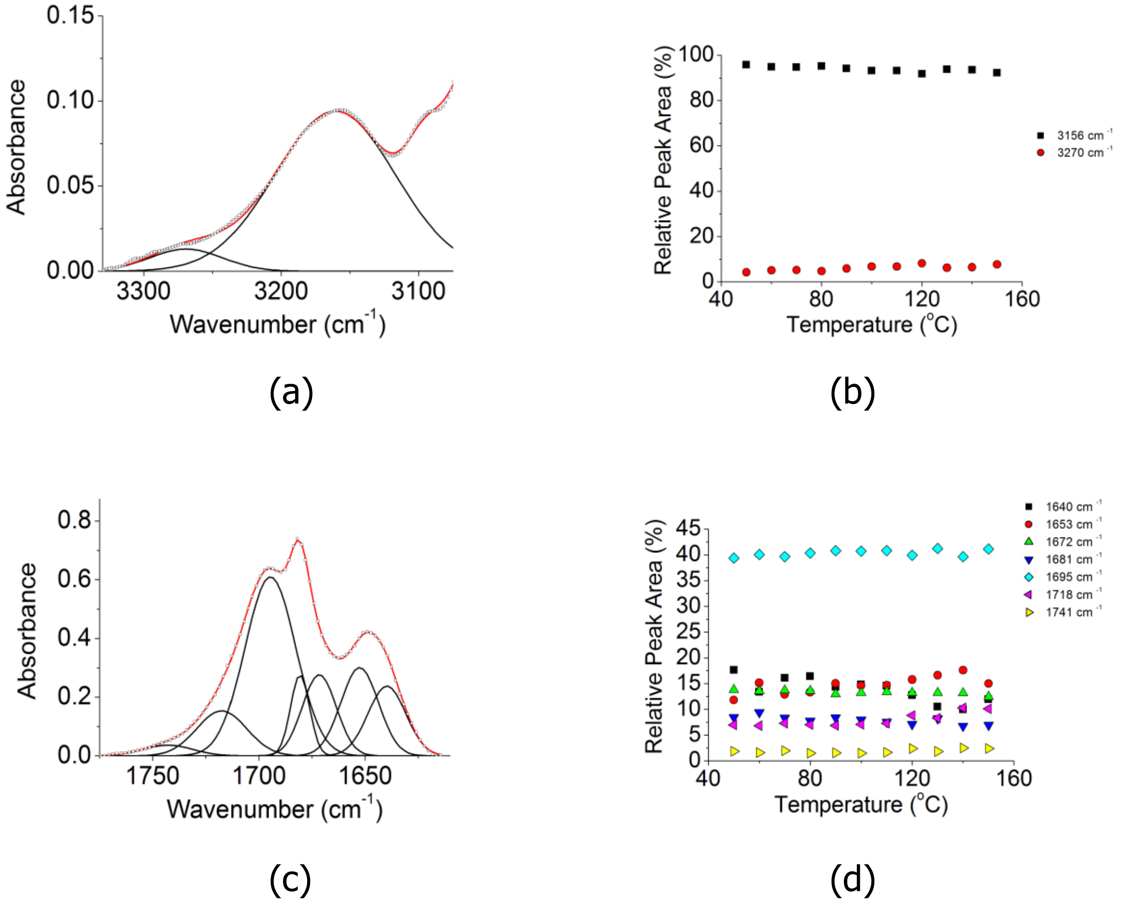

Figure S6. Temperature dependence of the FT-IR spectra of **tAZOi**, including the curves at $T = 150$°C and the deconvolution to several individual contributions, as well as the temperature dependence of the individual relative areas for the: **(a)**, **(b)** N-H st.; and **(c)**, **(d)** C=O *st.* regions.

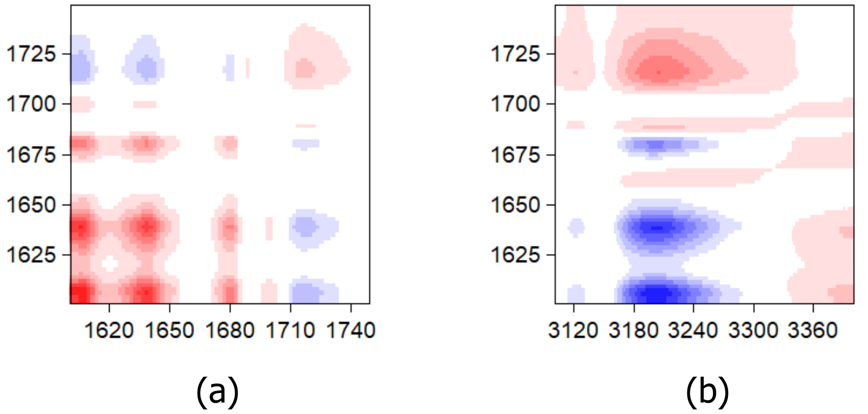

Figure S7. Synchronous 2D-IR plots corresponding to **tAZOi**, obtained on cooling from $T = 150$°C. **(a)** C=O stretching region; **(b)** cross-correlation between the C=O and N-H stretching regions. All axes display wavenumber, in cm^{-1}. The appearance of cross-correlations between the NH and C=O groups indicate temperature dependence of hydrogen bonding in this sample.

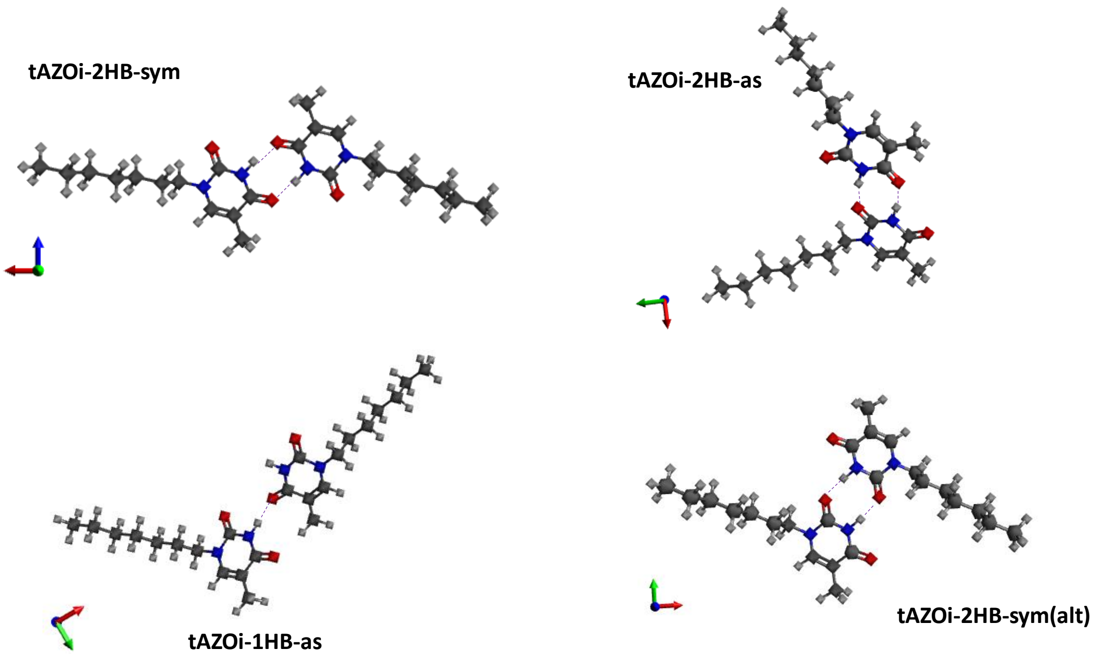

Figure S8. Dimers predicted by density functional theory calculations, DFT, corresponding to different tAZOi supramolecular assemblies. Dotted lines indicate hydrogen bonds.

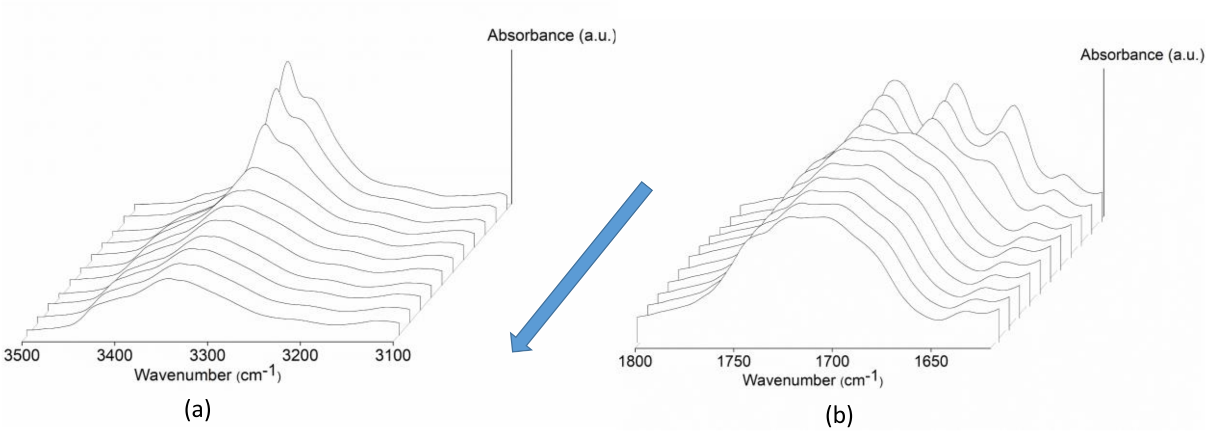

Figure S9. 3D-plots showing the temperature dependence of the FT-IR spectra of **DAP**, in the **(a)** N-H *st.* and **(b)** C=O *st.* regions. Arrow indicates the direction on cooling.

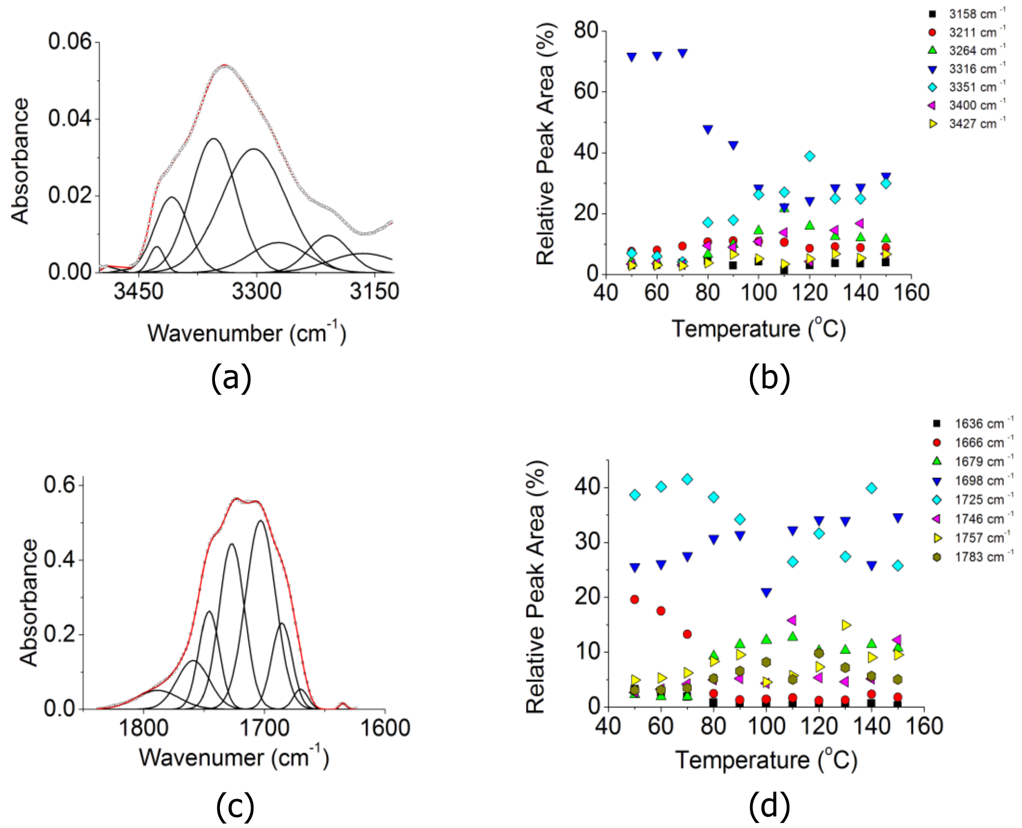

Figure S10. Temperature dependence of the FT-IR spectra of **DAP**, including the curves at $T = 150°C$ and the deconvolution to several individual contributions, as well as the temperature dependence of the relative individual areas for the: **(a)**, **(b)** N-H *st.*; and **(c)** , **(d)** C=O *st.* regions.

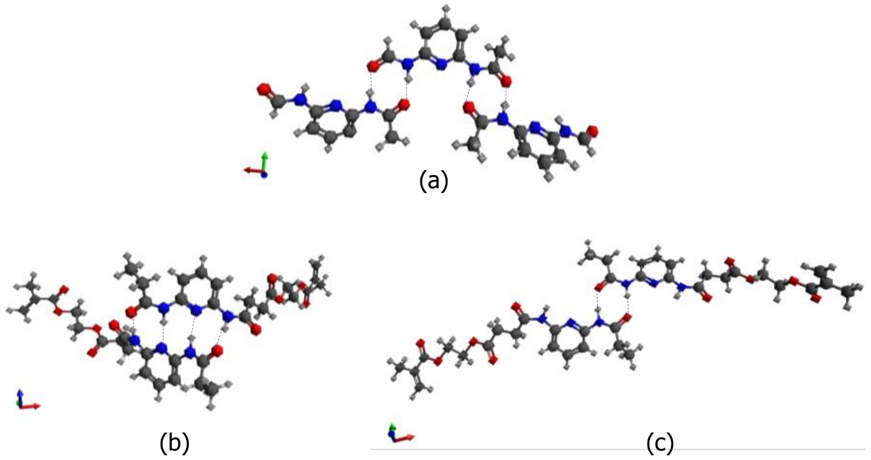

Figure S11. Dimers and trimers obtained by density functional theory calculations, DFT, corresponding to different DAP supramolecular assemblies: **(a) DAP3-2HB-as**, trimer with two double hydrogen bonds; **(b) DAP2-4HB-sym**, dimer with one quadruple hydrogen bond; **(c) DAP2-2HB-sym**, dimer with one double hydrogen bond. Dotted lines indicate hydrogen bonds.

Figures S12 to S15, show 3D plots corresponding to the temperature dependence of the FT-IR response of the mixtures, **DAP+dAZOi** and **DAP+ tAZOi**, as well as examples of fittings at high temperature and the corresponding evolution of the individual peaks areas on cooling.

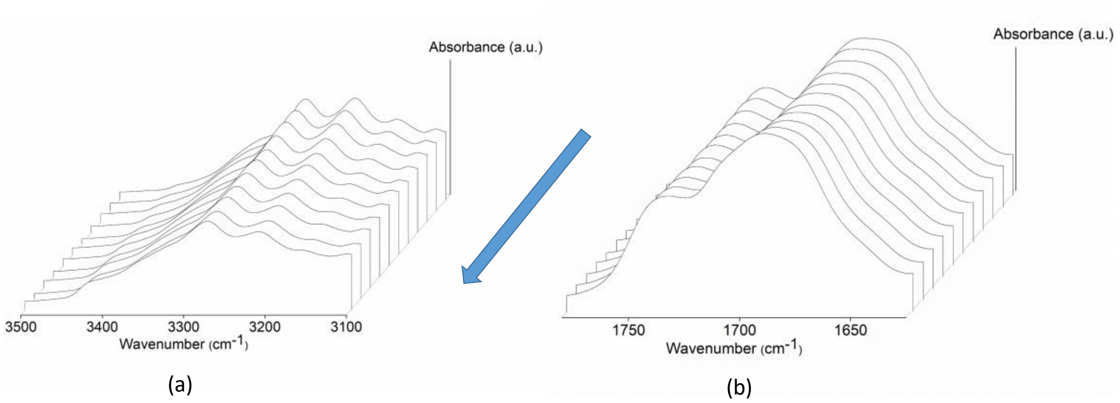

Figure S12. 3D-plots showing the temperature dependence of the FT-IR spectra of the mixture **DAP+ tAZOi**, in the **(a)** N-H *st.* and **(b)** C=O *st.* regions. Arrow indicates the direction on cooling.

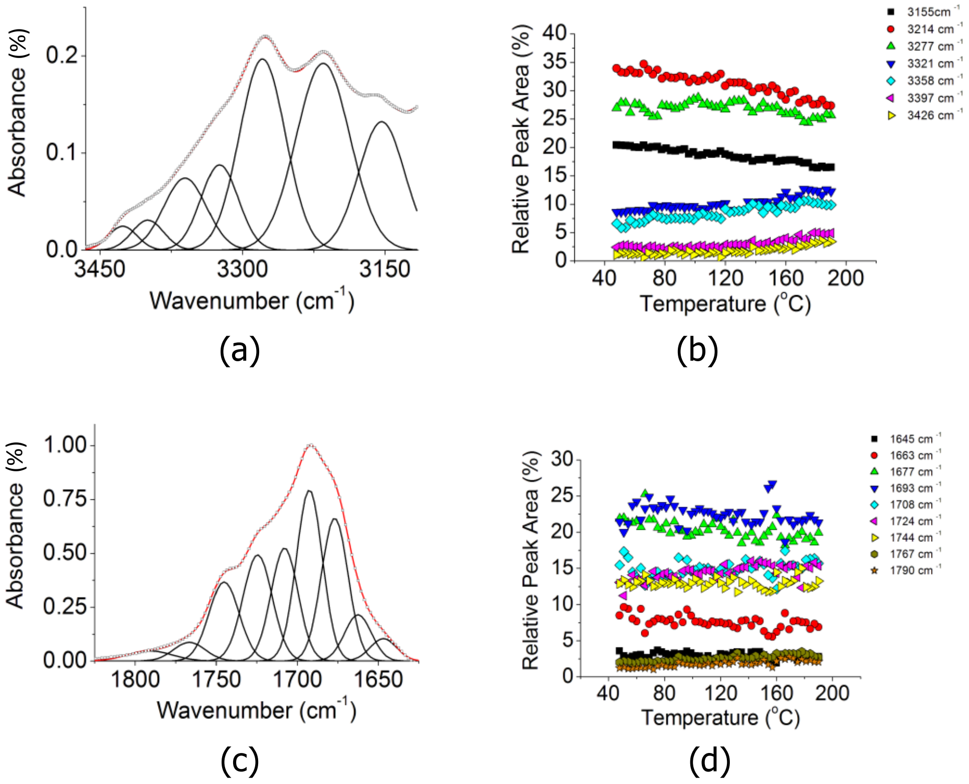

Figure S13. Temperature dependence of the FT-IR spectra corresponding to the mixture **DAP+ tAZOi**, including the curves at $T = 150°C$ and the deconvolution to several individual contributions, as well as the temperature dependence of the relative areas for the: **(a)**, **(b)** N-H *st.*; and **(c)** , **(d)** C=O *st.* regions.

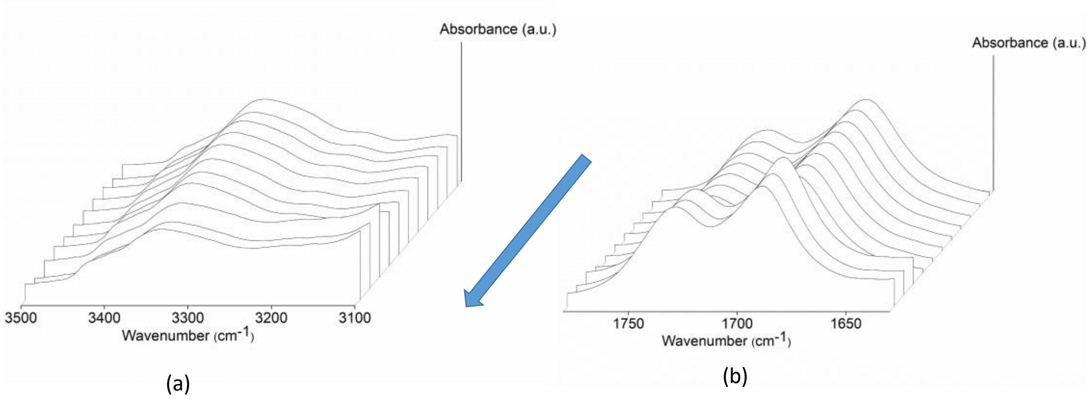

Figure S14. 3D-plots showing the temperature dependence of the FT-IR spectra of the mixture **DAP+dAZOi**, in the **(a)** N-H *st.* and **(b)** C=O *st.* regions. Arrow indicates the direction on cooling.

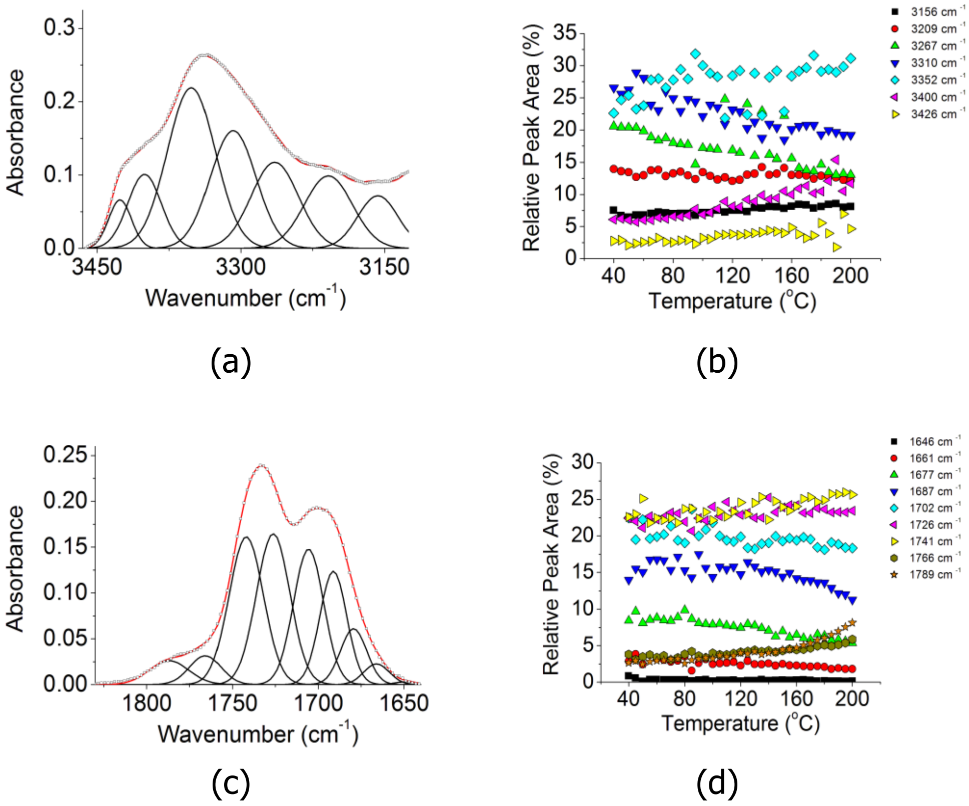

Figure S15. Temperature dependence of the FT-IR spectra corresponding to the mixture **DAP+dAZOi**, including the curves at $T = 150°C$ and the deconvolution to several individual contributions, as well as the temperature dependence of the relative areas for the: **(a)**, **(b)** N-H *st.*; and **(c)** , **(d)** C=O *st.* regions.

4. Additional tables.

Tables S1 to S4 show comparisons between the experimentally obtained maxima, and the values estimated by DFT, used to assign bands to individual contributions. Table S5 summarises the lengths of the hydrogen bonds obtained in the models.

Table S1. Maxima of the IR bands obtained experimentally for **dAZOi** (T = 150°C) and theoretical values predicted for their conformers obtained by DFT. HB: Hydrogen bonded, f: free.

**Contribution**	**Band maxima (cm^{-1})**		
**Experimental**			
**dAZOi**	1730, 1710	1694	1687
**Calculated DFT**			
**dAZOi-0HB (monomer)**	1725, f		
**dAZOi-2HB-as**	1728, f	1684, HB	
**dAZOi-2HB-sym**			1662, HB

Table S2. Experimental (T = 150°C) and theoretical (calculated by DFT) peaks observed in the N-H stretching vibration region for **tAZOi**. HB: hydrogen bonded, ad-HB: adjacent to hydrogen bonding, f: free.

**Contribution**		**Band maxima (cm^{-1}) - region**					
**Experimental**		**1760-1740**	**1725**	**1698**		**1679, 1666**	**3270, 3156**
**Calculated DFT**	**tAZO$_i$-0HB monomer**	1734 Urea, f		1717 Ring		1691 α,β-unsaturated, f	> 3600
	**tAZO$_i$-1HB-as open asymetric**	1741 Urea, ad-HB	1732 Urea, f	1710, 1706 Ring		1682 α,β-unsaturated, HB	3354, HB
	**tAZO$_i$-2HB-sym closed symmetric**	1731 α,β-unsaturated, ad-HB		1705 Ring		1678 Urea HB	
	**tAZO$_i$-2HB-sym(alt) closed, symmetric**	1737 Urea, ad-HB		1706 Ring		1675 α,β-unsaturated HB	3292-3150, HB
	**tAZO$_i$-2HB-as closed asymmetric**	1733 Urea, f α,β-unsaturated, ad-HB		1706 Ring	1699 Urea HB	1679 α,β-unsaturated HB	

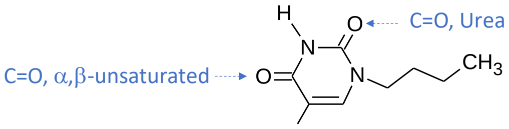

Table S3. Experimental ($T = 150°C$) and theoretical (calculated by DFT) band maxima peaks (cm^{-1}) observed in the N-H stretching vibration region for **DAP**.

**Experimental***	**Calculated DFT**		
**DAP**	**DAP2-4HB-sym**	**DAP3-2HB-as**	**DAP2-2HB-sym**
3427	-	3580	-
3400	-	-	3565
3351	-	3288	-
3316	3252	3262	3258
3264	3232	3240	-
3211	3195	3215	3207

Table S4. Experimental bands obtained for **DAP+tAZO**$_i$ (T = 150℃) and theoretical (**DAP●tAZOi-3HB**) peaks maxima (cm^{-1}) in the NH and C=O stretching vibration regions.

**N-H *st.* region**		**C=O *st.* region**		
**Experimental**	**Calculated DFT**	**Experimental**	**Calculated DFT**	
3426		1744	1754	tAZOi ring
3397		1724	1720	Ester
3358	-		1715	In DAP amide (adjacent to HB)
3321	3303 HB with Urea C=O	1708	1708	
3277	3275 HB with α,β unsaturated C=O	1693	1693	tAZOi (ring)
3214		1677	1679	
≤ 3155	2982 HB with Pyridine ring	1663		

Table S5. Lengths (Å) of the hydrogen bonds estimated in the models by DFT.

Conformer / HB	NH---C=O	NH---Pyr	OH---C=O	OH---OH	OH---Pyr
**Dimers (and trimer)**					
**DAP2-4HB-sym**	1.818	1.953			
**DAP2-2HB-sym**	1.810				
**DAP3-2HB-as**	1.819				
**tAZOi-2HB-sym(alt)**	1.798				
**tAZOi-2HB-as**	1.788				
**tAZOi-1HB-as**	1.815				
**tAZOi-2HB-sym**	1.777				
**dAZOi-2HB-as**			1.786	1.811	
**dAZOi-2HB-sym**			1.583		
**Complexes**					
**DAP•tAZOi-3HB**	1.886	1.797			
**DAP•dAZOi-2HB**	1.853				1.578
**Pyr•tAZOi-1HB**		1.771			
**Pyr•dAZOi-1HB**					1.668

5. Theoretical IR spectra calculated by DFT

Figures S16 to S28 show the theoretical spectra obtained in the gas phase, for all the species modelled by DFT.

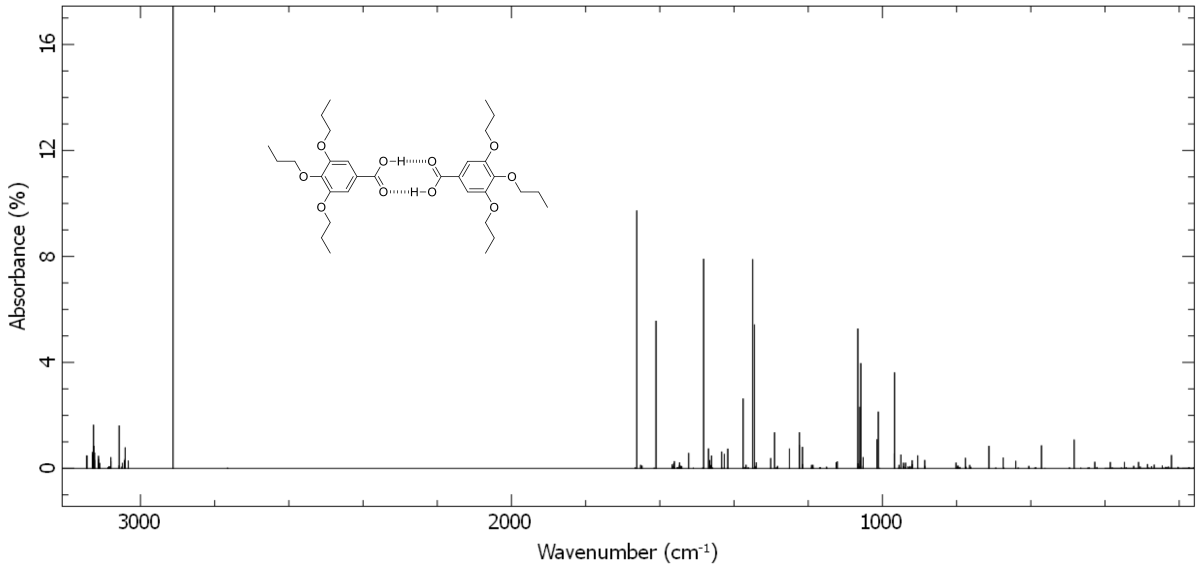

Figure S16. IR theoretical bands obtained by DFT for **dAZO$_i$-2HB-sym** in the gas phase.

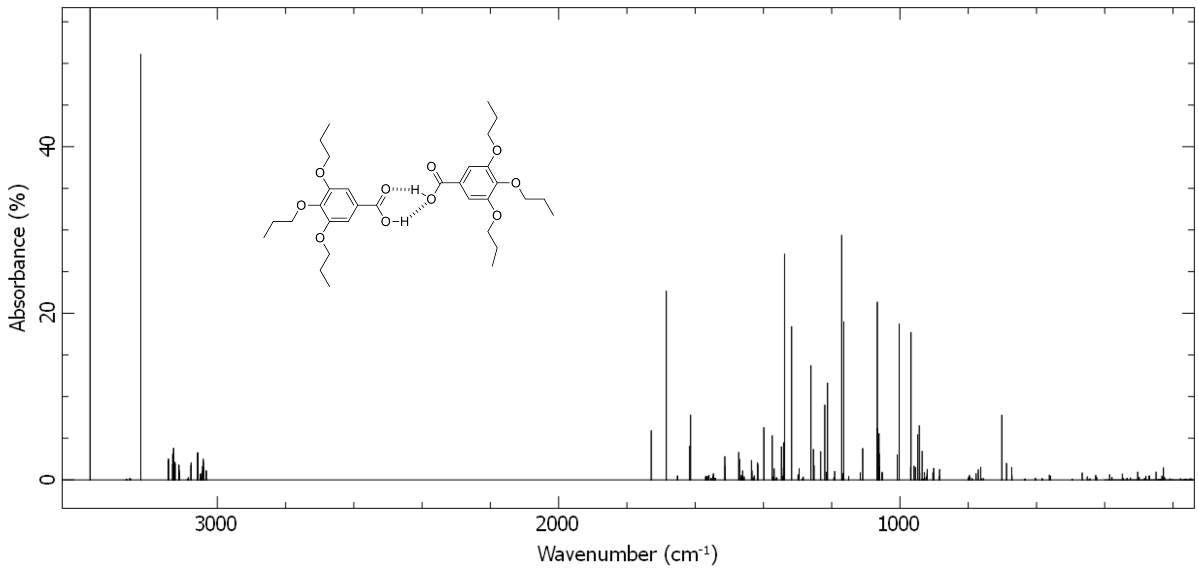

Figure S17. IR theoretical bands obtained by DFT for **dAZO$_i$-2HB-as** in the gas phase.

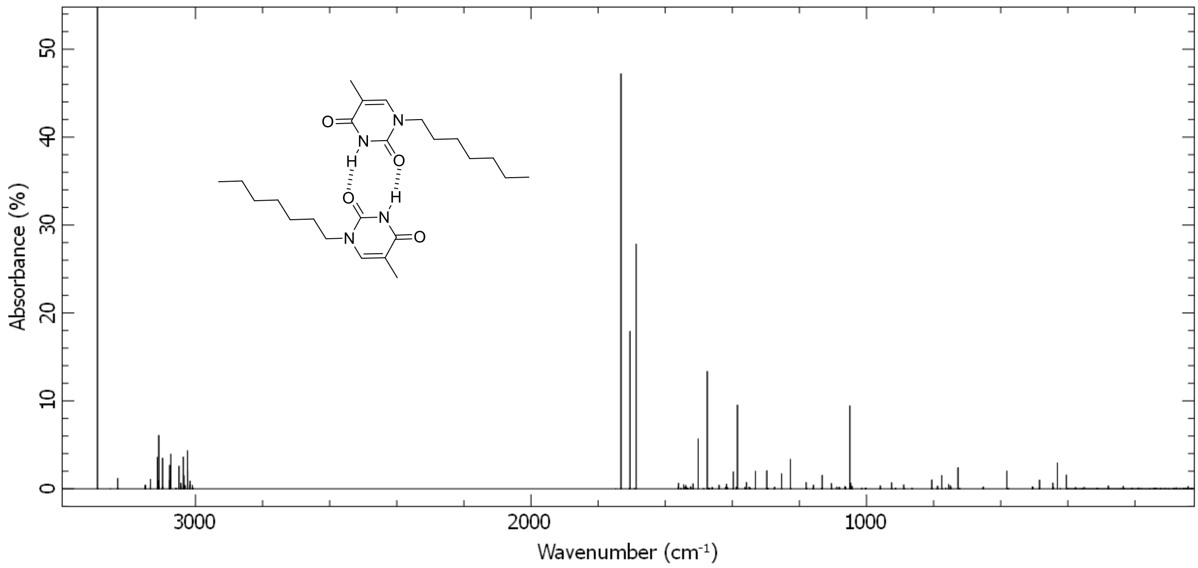

Figure S18. IR theoretical bands obtained by DFT for **tAZO$_i$-2HB-sym(alt)** in the gas phase.

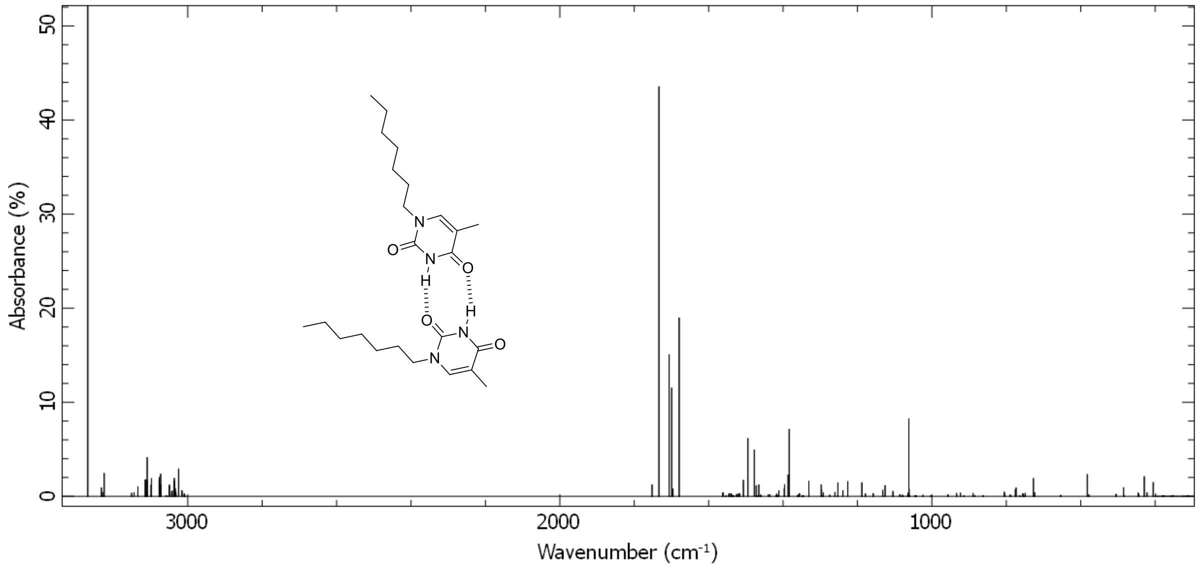

Figure S19. IR theoretical bands obtained by DFT for **tAZO$_i$-2HB-as** in the gas phase.

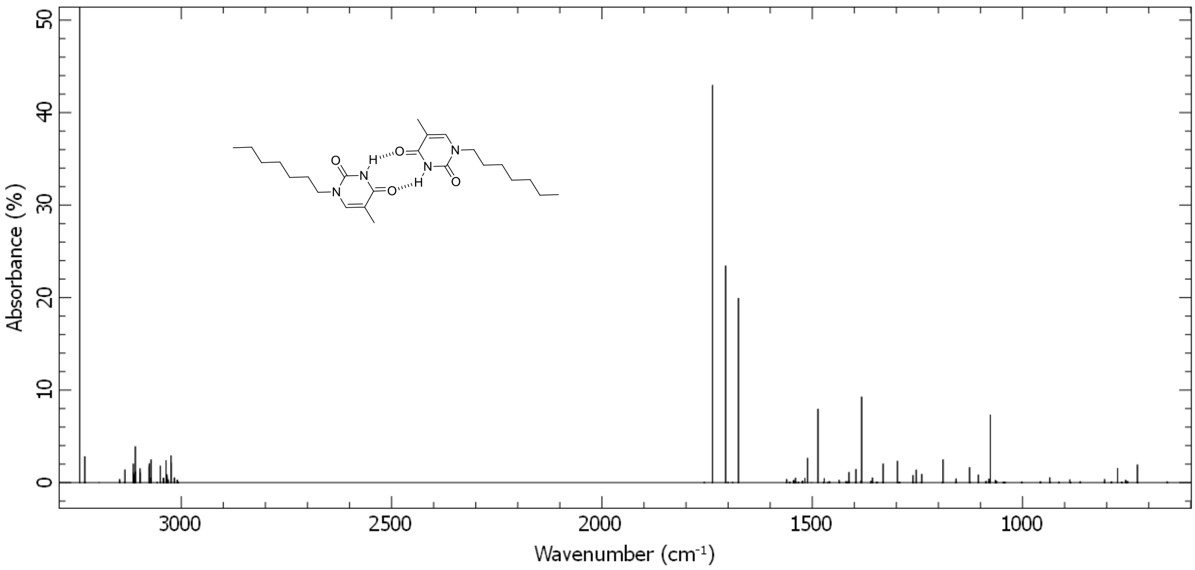

Figure S20. IR theoretical bands obtained by DFT for **tAZOi-2HB-sym** in the gas phase.

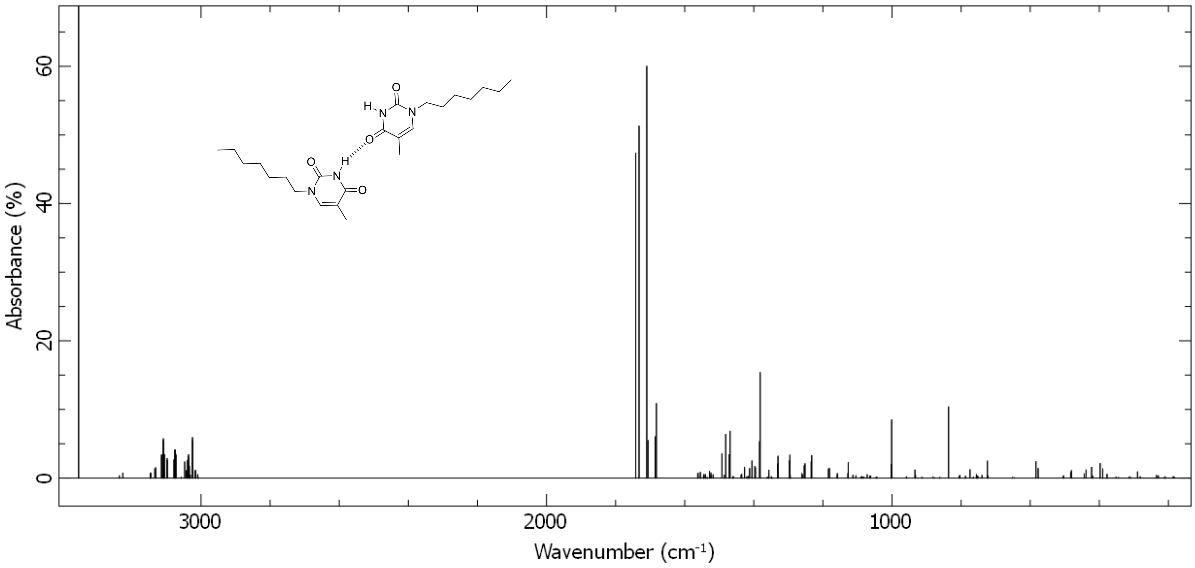

Figure S21. IR theoretical bands obtained by DFT for **tAZOi-1HB-as** in the gas phase.

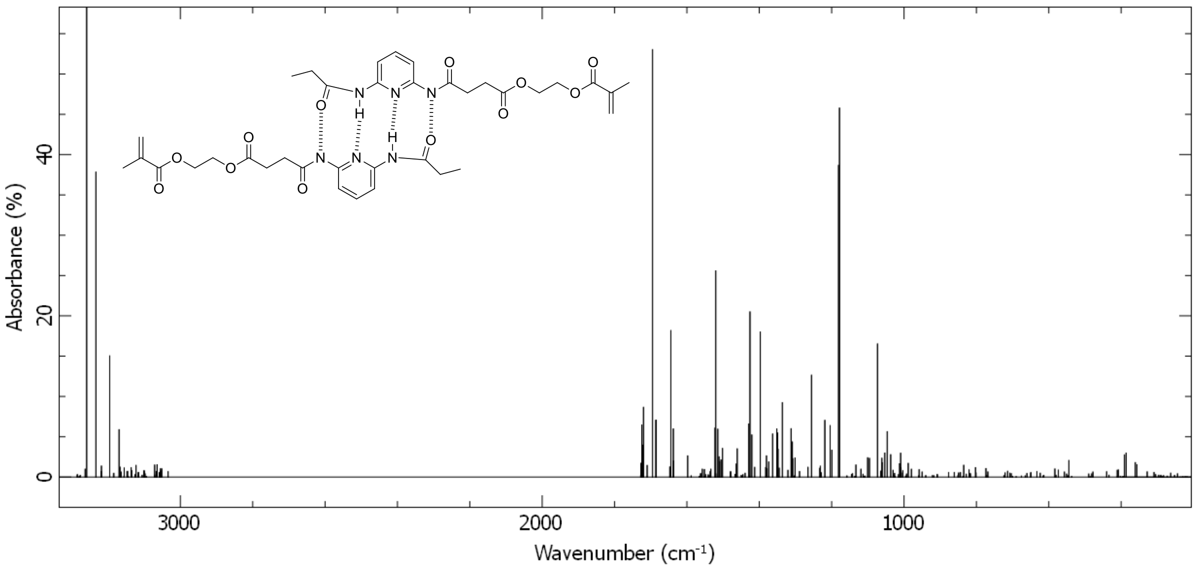

Figure S22. IR theoretical bands obtained by DFT for **DAP2-4HB-sym** in the gas phase.

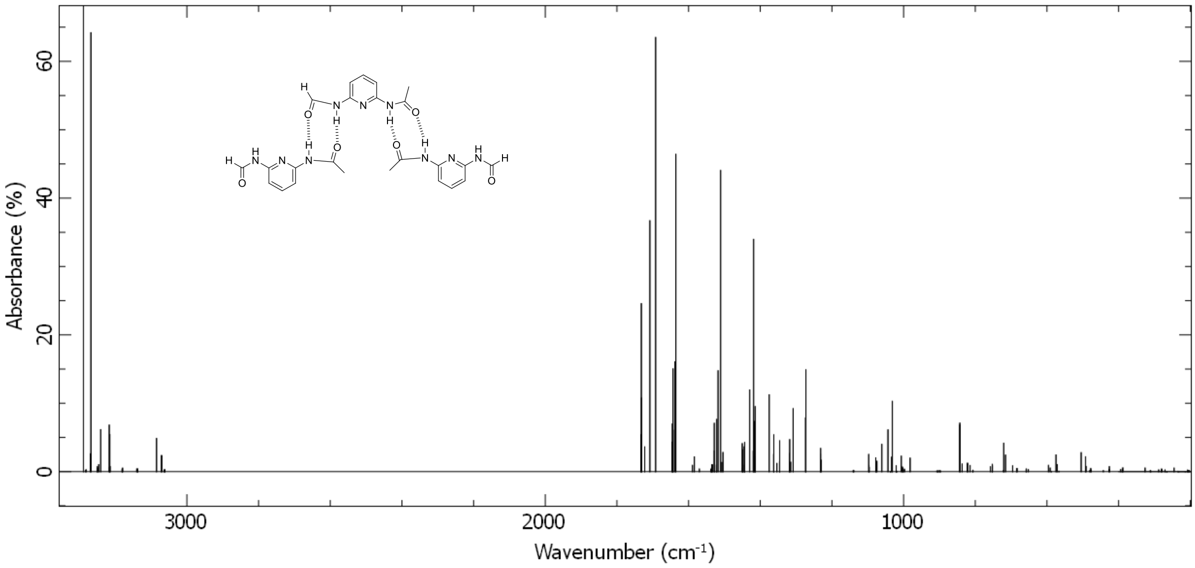

Figure S23. IR theoretical bands obtained by DFT for **DAP3-2HB-as** in the gas phase.

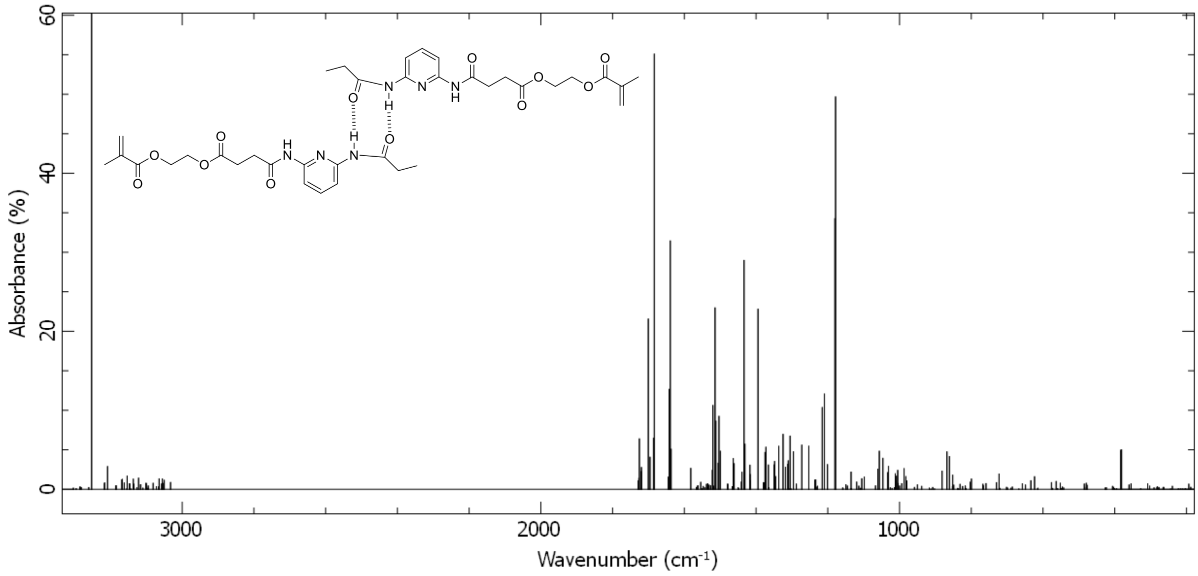

Figure S24. IR theoretical bands obtained by DFT for **DAP2-2HB-sym** in the gas phase.

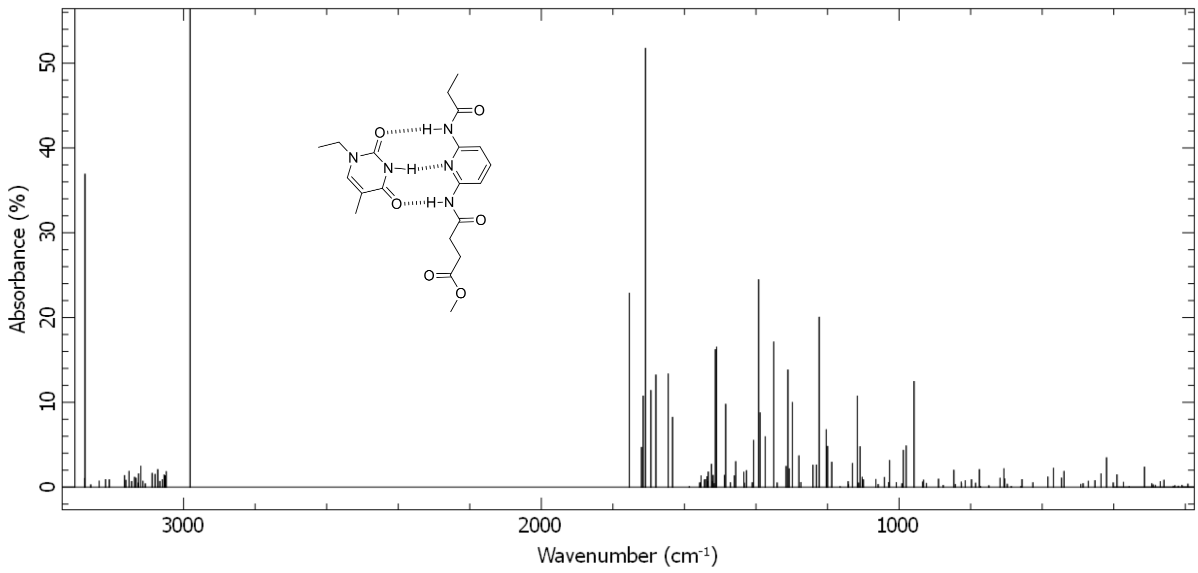

Figure S25. IR theoretical bands obtained by DFT for **DAP•tAZOi-3HB** in the gas phase.

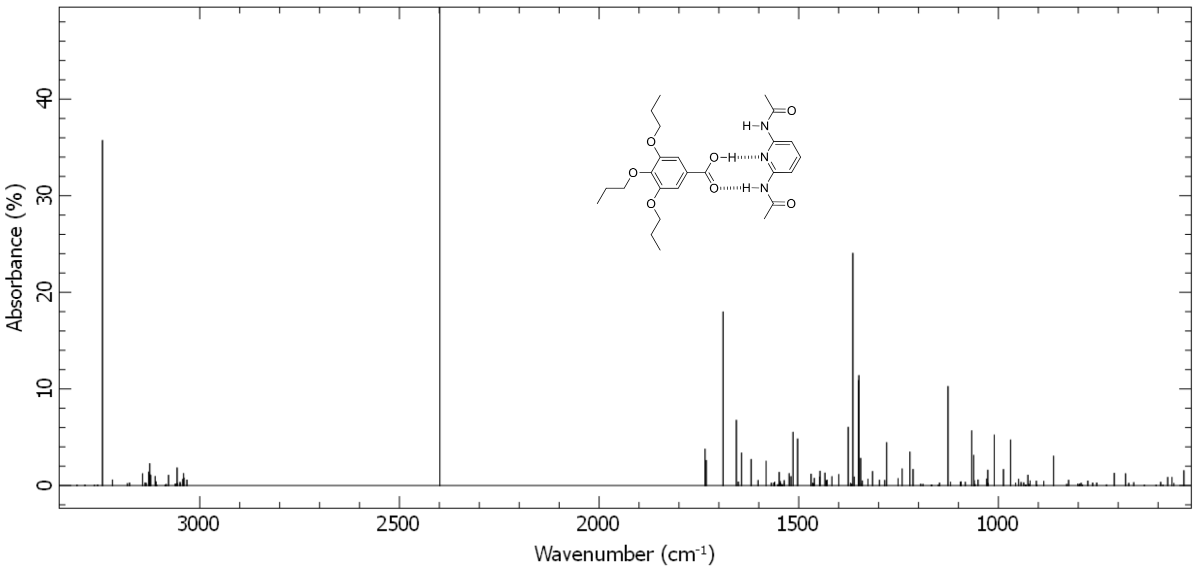

Figure S26. IR theoretical bands obtained by DFT for **DAP•dAZOi-2HB** in the gas phase.

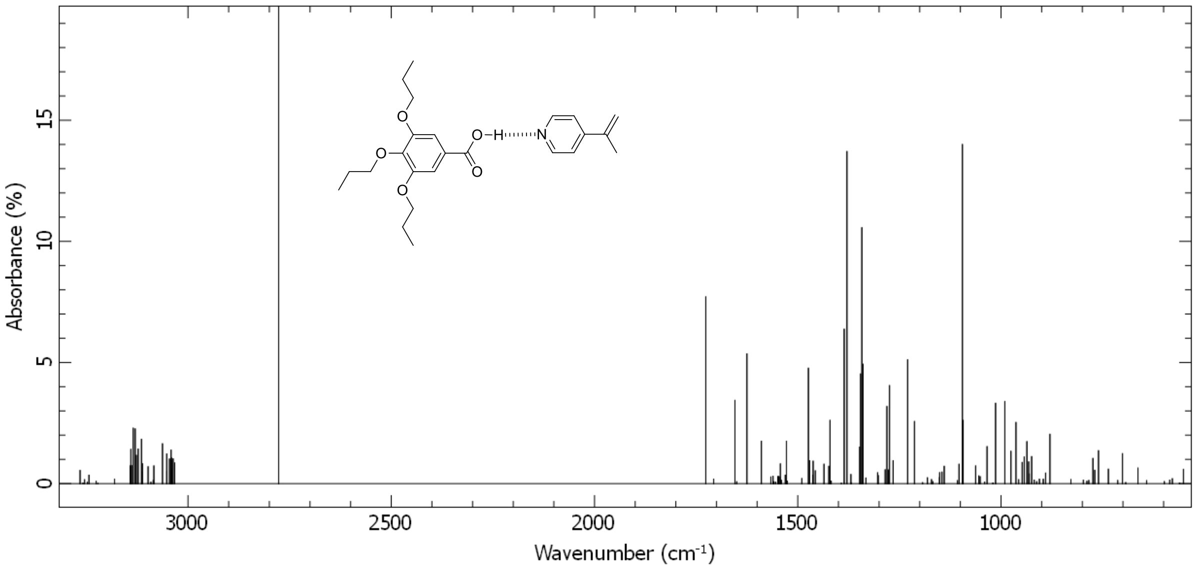

Figure S27. IR theoretical bands obtained by DFT for **Pyr•dAZOi-1HB** in the gas phase.

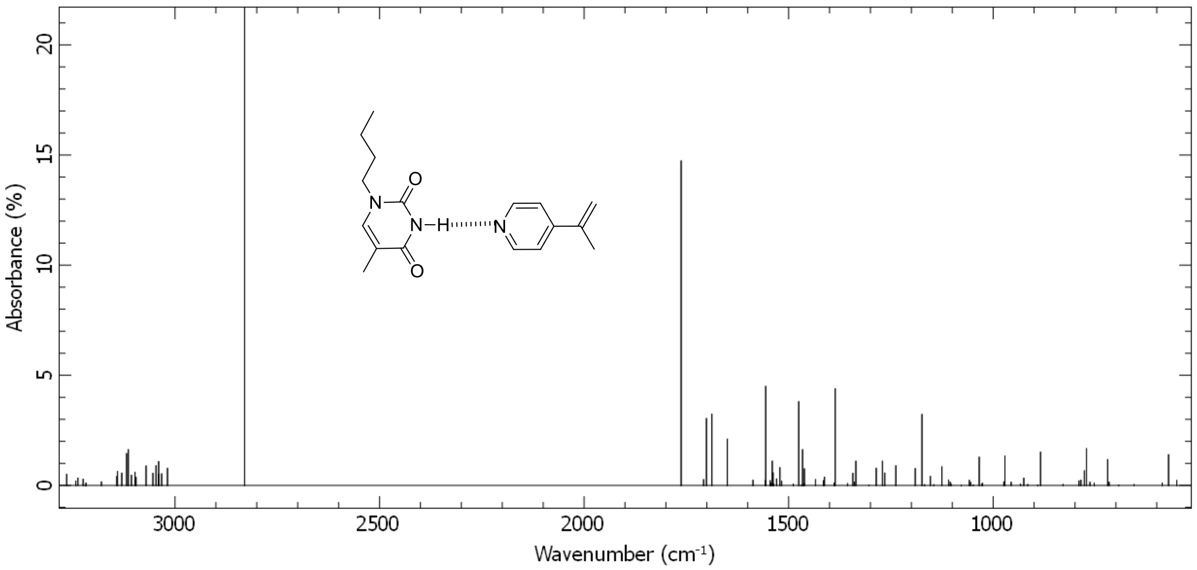

Figure S28. IR theoretical bands obtained by DFT for **Pyr•tAZOi-1HB** in the gas phase.

6. References

1. Concellon, A.; Blasco, E.; Pinol, M.; Oriol, L.; Diez, I.; Berges, C.; Sanchez-Somolinos, C.; Alcala, R. Photoresponsive polymers and block copolymers by molecular recognition based on multiple hydrogen bond *J. Polym. Sci. Pol. Chem.* **2014**, 52, 3173-3184. DOI: 10.1002/pola.27373.

2. Concellon, A.; Blasco, E.; Martinez-Felipe, A.; Martínez, J.L.; Sics, I.; Ezquerra, T.A.; Nogales, A.; Pinol, M.; Oriol, L. Light-responsive self-assembled materials by supramolecular post-functionalization via hydrogen bonding of amphiphilic block copolymers. *Macromolecules*, **2016**, 49, 7825-7836. DOI: 10.1021/acs.macromol.6b01112.

3. Noda I. Frontiers of two-dimensional correlation spectroscopy. Part 1. New concepts and noteworthy developments. *J. Mol. Struct.* **2014**, 8, 1069, 3-22. DOI: 10.1016/j.molstruc.2014.01.025.

4. Noda I. Determination of two-dimensional correlation spectra using the Hilbert transform. *Appl. Spectrosc.* **2000**, 54(7), 994-999. DOI: 10.1366/0003702001950472.

5. Noda I. Recent developments in two-dimensional (2D) correlation spectroscopy. *Chin. Chem. Lett.* **2015**, 26, 167-172. DOI: 10.1016/j.cclet.2014.10.006.

6. Noda I. Two-dimensional codistribution spectroscopy to determine the sequential order of distributed presence of species. *J. Mol. Struct.* **2014**, 1069, 50-59. DOI: 10.1016/j.molstruc.2014.01.024.

7. Frisch, M. J.; Trucks, G.W.; Schlegel, H.B.; Scuseria, G.E.; Robb, M.A.; Cheeseman, J.R.; Scalmani, G.; Barone, V.; Mennucci, B.; Petersson, G.A.; et al, inventors. Gaussian (revision D. 01) Gaussian Inc, Wallingford, CT. US patent . **2009**.

8. Lii J; Ma B; Allinger N. Importance of selecting proper basis set in quantum mechanical studies of potential energy surfaces of carbohydrates. J. Comp. Chem. **1999**, 20, 1593-1603. DOI: https://doi.org/10.1002/(SICI)1096-987X(19991130)20:15<1593::AID-JCC1>3.0.CO;2-A.

9. Kim KK; Jordan KD. Comparison of Density Functional and MP2 Calculations on the Water Monomer and Dimer *J. Phys. Chem.*, **1994**, 98, 10089–10094., DOI: 10.1021/j100091a024.

10. Frisch MJ; Del Bene JE; Binkley JS; Schaefer HF. Extensive theoretical studies of the hydrogen bonded complexes $(H_2O)_2$, $(H_2O)2H^+$, $(HF)_2$, $(HF)_2H^+$, F_2H^-, and $(NH_3)_2$ *J. Chem. Phys.* **1986**, 84, 2279. DOI: 10.1063/1.450390.

11. Borba A; Albrecht M; Gomez-Zavaglia A; Lapinski L; Nowak MJ; Suhm MA; Fausto R. Dimer formation in nicotinamide and picolinamide in the gas and condensed phases probed by infrared spectroscopy *Phys. Chem. Chem. Phys.* **2008**, 10, 7010–7021, DOI: 10.1039/b810002.

12. Martinez-Felipe, A.; Cook, AG.; Wallage, M.J.; Imrie, C.T. Hydrogen bonding and liquid crystallinity of low molar mass and polymeric mesogens containing benzoic acids: A variable temperature Fourier transform infrared spectroscopic study. Phase Transit. **2014**, 87, 1191-1210. DOI: 10.1080/01411594.2014.900556.
